# Genetic diversity of North American captive-born gorillas (*Gorilla gorilla gorilla*)

**DOI:** 10.1002/ece3.422

**Published:** 2013-01-10

**Authors:** Noah D Simons, Ronald S Wagner, Joseph G Lorenz

**Affiliations:** 1 Primate Behavior Program, Central Washington UniversityEllensburg, Washington; 2 Department of Biological Sciences, Central Washington UniversityEllensburg, Washington; 3 Department of Anthropology and Museum Studies, Central Washington UniversityEllensburg, Washington

**Keywords:** Captive populations, conservation genetics, gorilla, microsatellite

## Abstract

Western lowland gorillas (*Gorilla gorilla gorilla*) are designated as critically endangered and wild populations are dramatically declining as a result of habitat destruction, fragmentation, diseases (e.g., Ebola) and the illegal bushmeat trade. As wild populations continue to decline, the genetic management of the North American captive western lowland gorilla population will be an important component of the long-term conservation of the species. We genotyped 26 individuals from the North American captive gorilla collection at 11 autosomal microsatellite loci in order to compare levels of genetic diversity to wild populations, investigate genetic signatures of a population bottleneck and identify the genetic structure of the captive-born population. Captive gorillas had significantly higher levels of allelic diversity (*t*_7_ = 4.49, *P* = 0.002) and heterozygosity (*t*_7_ = 4.15, *P* = 0.004) than comparative wild populations, yet the population has lost significant allelic diversity while in captivity when compared to founders (*t*_7_ = 2.44, *P* = 0.04). Analyses suggested no genetic evidence for a population bottleneck of the captive population. Genetic structure results supported the management of North American captive gorillas as a single population. Our results highlight the utility of genetic management approaches for endangered nonhuman primate species.

## Introduction

Wild populations of western lowland gorillas (*Gorilla gorilla gorilla*) are facing dramatically declining numbers, as high as 80% over three generations, as a result of habitat destruction and fragmentation, the illegal bushmeat trade, and diseases such as Ebola (Walsh et al. 2008). Due to rapidly increasing human encroachment into gorilla habitat, captive gorilla populations represent an important aid to long-term conservation as a representative species communicating conservation issues in great ape range countries to the public. The captive collection of gorillas in North America began over 100 years ago with wild individuals imported from Africa; however, since coming under protection of the Convention on International Trade in Endangered Species of Wild Fauna and Flora (CITES) in 1975 there have been no new wild gorillas added to zoos (Nsubuga et al. 2010). Further, they were designated as critically endangered in 2007 (Walsh et al. 2008).

In the wild, western gorillas (*Gorilla gorilla diehli, G. g. gorilla*) are separated from eastern gorillas (*Gorilla beringei beringei, Gorilla beringei graueri*) by the Congo River in central Africa. Eastern gorillas are listed as endangered at the species level and the mountain gorilla subspecies (*G. b. beringei*) is listed as critically endangered (Walsh et al. 2008). With the exception of the mountain gorilla populations in Bwindi and Karisoke, which have seen a population increase and stabilization in the last 20 years due to “extreme” conservation efforts, other wild populations continue to decline (Guschanski et al. 2009; Robbins et al. 2011; Gray et al. 2007). Captive populations, with no influx of wild individuals, may potentially face the same problems regarding loss of genetic diversity as small wild populations without appropriate genetic management (Ballou and Lacy 1995). The captive North American population of ∼370 individuals has the potential to lose genetic diversity through random genetic drift if not properly managed as a single population, thus it is critical to have a breeding program that aims to maximize genetic diversity in order to avoid inbreeding depression. The gorilla Species Survival Plan (SSP) goals for maintaining genetic diversity are to maintain >90% of the genetic diversity of the previous generation, over 100 years, following Frankham et al. (2002).

Breeding protocols of the North American Gorilla SSP include determining breeding pairs based on pedigree information (Nsubuga et al. 2010). However, accurate pedigree determination can be challenging because importation records of wild caught gorillas can be inaccurate. For example, Nsubuga et al. (2010) discovered that a known breeding pair consisted of first order relatives from the founder population of wild-born captive gorillas. This is not an error of the zoos but reflects the difficulty of establishing kinship relationships in the absence of genetic data. The use of molecular markers, particularly microsatellite panels, has been shown to be a highly effective genetic management tool in nonhuman primates (Deinard and Kidd 2000; Meier et al. 2000; Kanthaswamy et al. 2006; Perwitasari-Farajallah et al. 2010). Microsatellites are the marker of choice for genetic management for a number of reasons including high polymorphic information content and gene diversity. They are also effective at determining individual genetic identity and parentage exclusion (Kanthaswamy et al. 2006). These are particularly true for microsatellites with a tetranucleotide repeat motif, which are more reliably characterized than dinucleotide repeats (Kanthaswamy et al. 2006). Genotyping individuals at polymorphic, neutral markers, such as microsatellites, and determining breeding pairs based on lowest mean kinship can help maximize genetic diversity in populations (Ballou and Lacy 1995).

We investigated genetic diversity of the North American captive-born population of gorillas using a well-characterized panel of microsatellite loci. We compared this new data set to previously collected data from wild gorillas as well as the wild-born founder population of captive North American gorillas to compare genetic diversity and investigate signatures of a genetic bottleneck. Because of high levels of genetic diversity and wide range of individuals in the wild-born founder population, we predicted that the genetic variation maintained in the captive-born population would be greater than that found in small wild populations of both western and eastern gorillas. Because of high levels of genetic diversity in the founder population, as a result of having multiple wild source populations (Nsubuga et al. 2010), we predicted a reduction in genetic diversity in the captive-born population compared to the wild-born founder population. Lastly, based on the long generation time of gorillas and the relatively short time in captivity, we predicted captive-born gorillas, which are all western lowland gorillas, will cluster with wild western lowland gorillas (WWLG) to the exclusion of less related cross river gorillas and eastern gorillas, but that those clusters will reflect ancestral gene-flow between cross river and western lowland gorillas.

## Methods

### Samples

DNA samples for captive western lowland gorillas (*N* = 26) were obtained from fibroblast cell lines located in the Integrated Primate Biomaterials and Information Resource collection at the Coriell Institute for Medical Research.

Comparative datasets for this study came from five wild populations of gorillas and one founder population of the captive North American western lowland gorillas (Nsubuga et al. 2010). Included in the wild populations were Cross River gorillas (*G. g. diehli*) from the Cameroon-Nigeria border (Bergl 2006), and two populations of western lowland gorillas (*G. g. gorilla*) from Loango National Park, Gabon, and Mondika in the Central African Republic (Bradley et al. 2004; Bergl et al. 2008; Arandjelovic et al. 2010, Fig. 1; respectively). The two western lowland gorilla populations were combined using the weighted mean of diversity measures and are referred to as wild western lowland gorillas. Eastern gorilla populations included two populations of mountain gorillas (*G. b. beringei*) from Bwindi Impenetrable Forest, Uganda and Volcanoes National Park in Virunga Mountains, Rwanda (Bradley et al. 2005; Nsubuga et al. 2008; Fig. 2). Based on sample sizes and sampling ranges we consider all four populations, and the founding population (WWLG, *N* = 131; Cross River, *N* = 71; Bwindi, *N* = 77; Virunga, *N* = 92; Founders, *N* = 79) to be small, comparable populations.

**Figure 1:**
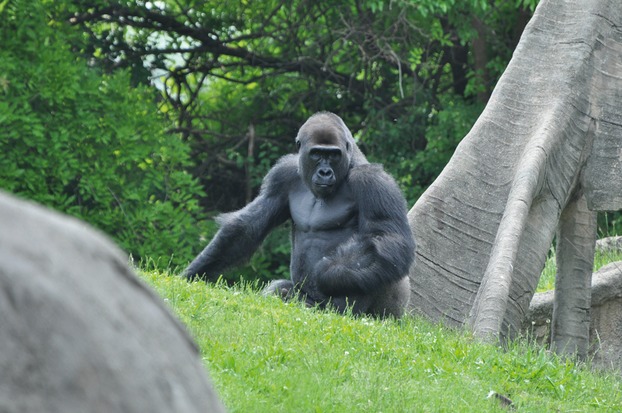
Western lowland gorilla (*Gorilla gorilla gorilla*). Photo Credit: Nelson Ting.

**Figure 2:**
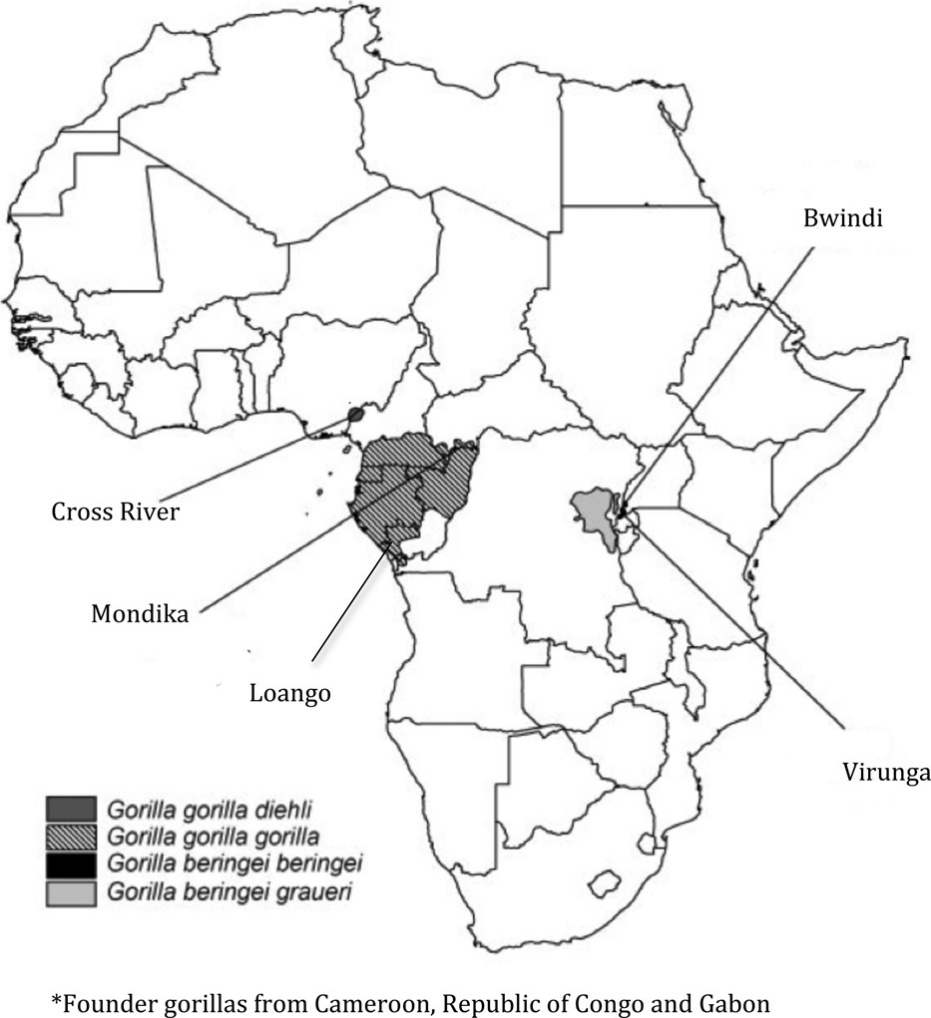
Distribution map of populations included in the study. Adapted from Bergl et al. (2008) *Founder gorillas from Camerron, Republic of Congo and Gabon.

### DNA amplification and genotyping

Multilocus genotypes from 11 polymorphic autosomal microsatellite loci were produced for 26 individual gorillas. Microsatellite loci included D1s550, D2s1326, D5s1470, D4s1627, D5s1457, vWF, D16s2624, D8s1106, D10s1432, D2s2204, and D7s817 (Primers from Bradley et al. 2000). This panel of microsatellite loci was chosen based on the availability of comparable datasets in the literature. All loci are tetranucleotide repeats with the exception of D5s1470, which is a tetranucleotide repeat with a 2 bp indel.

PCR reactions were carried out with the following: 10 μL (5 U/μL) AmpliTaq Gold (Applied Biosystems, Foster City, CA), 0.4 μL (10 μmol/L) of forward and reverse primers, 7.7 μL dH_2_O and 1.5 μL (10–30 ng/μL) DNA template for a final volume of 20 μL. Thermocycler protocol included a 10 min initial denaturing at 95°C, followed by 40 cycles of 94°C denaturing for 30 sec, 55–60°C annealing for 30 sec (Table 1), 72°C extension for 30 sec, followed by a final extension of 72°C for 30 min. Forward primers were labeled at the 5′ end with IR Dye 700 fluorescent label. Amplicons were separated by gel electrophoresis on a LiCor 4300 DNA Analyzer using 6.5% KB Plus gel matrix (LiCor Biosciences, Lincoln, NE). Alleles were scored using an IR Dye 700 internal size standard in SagaGT genotyping software (LiCor Biosciences). Genotype data was checked for allelic dropout and null alleles using CERVUS 3.0.3 (Marshall et al. 1998; Kalinowski et al. 2007).

**Table 1:** Allelic diversity and heterozygosity of captive-born gorillas

Captive-Born	*NA*	*AR*	*AE*	*H*_I_	*H*_O_	*H*_E_
D16s2624	05.00	4.99	3.59	–	0.73	0.72
D10s1432	07.00	6.80	5.18	–	0.80	0.80
D7s817	09.00	8.65	4.70	–	0.95	0.78
D7s2204	07.00	6.72	2.53	–	0.79	0.60
D4s1627	06.00	5.99	5.56	–	0.96	0.82
D2s1326	10.00	9.58	7.23	–	0.96	0.86
D1s550	07.00	6.74	3.93	–	0.80	0.74
vWF	07.00	6.77	4.28	–	0.88	0.76
Mean	07.25	7.03	4.63	0.86	0.87	0.76

*NA*, number of alleles; *AR*, allelic richness; *AE*, number of effective alleles; *H*_I_, individual heterozygosity; *H*_O_, observed heterozygosity; *H*_E_, Nei's expected heterozygosity.

### Genetic diversity comparisons

The captive-born gorilla dataset was examined for evidence of deviation from Hardy–Weinberg equilibrium and linkage disequilibrium in GENEPOP 4.0.9 (Rousset 2008). Genetic variation in the captive-born population was compared to the wild-born population of founder gorillas and four populations of wild gorillas using measures of allelic diversity and heterozygosity. Of the 11 microsatellites used, a subset of eight loci was used for inter-population comparisons based on the availability of comparable datasets in the literature. The number of alleles (*NA*), and number of effective alleles (*AE*; Kimura and Crow 1964), were calculated for the captive-born and Loango dataset (Arandjelovic et al. 2010) in GenAlEx 6.01 (Peakall and Smouse 2006). Number of effective alleles is a measure of the evenness of the allele frequency distribution averaged over all loci. This measure is suited for comparing populations with differing numbers of alleles. Allelic richness (*AR*), a measure of alleles which controls for differences in sample size, was calculated for the captive-born and Loango populations in FSTAT 2.9.3.2 (Goudet 2001). Captive-born and wild populations were evaluated for differences in allelic diversity (*NA, AR, AE*) using *t*-tests. Levels of observed heterozygosity (*H*_o_), and expected heterozygosity (*H*_e_), were calculated in GenAlEx 6.01 (Peakall and Smouse 2006). Mean individual heterozygosity (*H*_I_), was calculated similar to Nsubuga et al. (2008), as the mean number of heterozygous loci for each gorilla, divided by the total number of loci. Levels of heterozygosity in the captive-born population were tested for differences among the founding population and wild populations using *t*-tests following Bergl et al. 2008; Archie 1985; Nei 1987). Statistical significance was set at α = 0.05 and corrected for multiple comparisons using the Holm's sequential Bonferroni adjustment (Holm 1979; Jaccard 1998). The Holm's sequential method is a more sophisticated correction that controls for inflation of the Type I error rate while also maintaining statistical power (Kromery and Dickson 1995). Adjusted levels of alpha ranged from 0.003 to 0.05. The statistical significance of stated *P* values are relative to adjusted levels of alpha.

### Genetic structure and population assignment

Genetic population structure in the captive-born population of gorillas was inferred using STRUCTURE 2.3.3 (Pritchard et al. 2000). For structure analysis, a subset of five loci was used (D8s1106, D16s2624, vWF, D5s1457, D1s550) based on availability of genotype datasets for comparative populations (Loango, Cross River, Virungas). This subset was used due to the Loango dataset containing genotypes from nested primers for five loci, which were removed. Due to differences is base-calling, the Loango dataset appeared to be called 2 bp below the other three populations for the remaining five loci. When the Loango dataset was corrected for this the allele frequency distribution matched for all four populations and the datasets were compatible. The program STRUCTURE uses a Bayesian model-based clustering method to infer population genetic structure under the assumption of *K* clusters, where *K* is the number of individual clusters or populations. In order to determine the optimal number of *K* for this dataset, 10 independent replicates were run for values of *K* = 1–5. For each replicate within each *K* value, tests were run with 100,000 burn-in steps followed by 1,000,000 Markov Chain Monte Carlo replications. All tests were run under an admixture model with correlated allele frequencies. The log-likelihood [ln *P*(*D*)] was averaged over independent runs for each value of *K*. Runs of high *K* values can potentially increase the posterior probability as well as variance between independent runs, leading to an overestimation of *K* (Rosenberg et al. 2001; Nsubuga et al. 2008). Following the method of Evanno et al. (2005), we used the ad hoc test statistic *ΔK*, which is the second order rate of change in ln *P*(*D*) across consecutive values of *K*. The use of *ΔK* to identify breakpoints in the dataset results in the true value of *K* being that with the greatest *ΔK*.

Population assignment tests were carried out with the four datasets used for inferring genetic population structure (captive-born, Loango, Cross River, Virungas) in order to assess whether the captive-born population would cluster with the Loango population, which is the closest population to founders as well as being the same sub-species, to the exclusion of the Cross River and Virungas. Assignment tests were carried out in GenAlEx 6.01 (Peakall and Smouse 2006). Pairwise population assignment tests were also conducted among captive-born population and Cross River, Loango and Virunga populations. Assignment tests were frequency-based following Paetkau et al. (1995). Assignments tests were based on the log-likelihood value of genotype frequency over all loci for each population. Individuals are then assigned to the population with highest log-likelihood.

### Demographic history

Signatures of a genetic bottleneck were tested in the captive-born population using the BOTTLENECK program (Piry et al. 1999). Because the mutation model for these microsatellite loci is not known, we used a two-phase mutation model (TPM), which combines the infinite alleles model (IAM) and step-wise mutation model (SMM). The TPM accounts for the unlikelihood that microsatellite loci will precisely follow either a strict SMM or IAM (Di Rienzo et al. 1994; Piry et al. 1999). Following the method of Weckworth et al. (2005) we performed runs with step-wise changes in the contribution of SMM to the TPM of 70%, 75%, 80%, 85% and 90%. Additionally, we ran the test under a strict IAM and SMM separately. Three tests were used to assess significance in the difference between *H*_E_ and *H*_EQ_, where *H*_E_ is the expected heterozygosity, assuming mutation-drift equilibrium, and *H*_EQ_ is a coalescent-based estimate of heterozygosity based on the observed *NA* (Piry et al. 1999). Tests used for significant differences in *H*_EQ_ and *H*_E_ were Wilcoxon sign-rank tests, sign test and standardized differences test.

## Results

### Genetic diversity in captive-born gorillas

No evidence of null alleles or allelic dropout was observed in the captive-born dataset. In both the captive-born gorillas (D4s1627) and Loango (vWF), a single locus was observed to deviate from Hardy–Weinberg equilibrium. Deviations that have been previously described in Cross River (D5s1470 and D8s1106), Bwindi and Virunga populations (D1s550 and D4s1627, respectively) were attributed to the inclusion of related individuals in the sample (Bergl et al. 2008). In those datasets it was shown that when closely related individuals were removed, those loci no longer deviated from equilibrium (Lukas et al. 2004; Bradley et al. 2005). Because the captive-born dataset includes closely related individuals, including three pairs of full siblings and two pairs of half siblings, we followed Bergl et al. (2008) in treating all loci as though they were in equilibrium and independent.

The captive-born population showed relatively high levels of both allelic diversity and heterozygosity measures (Table 1). The captive-born gorilla population had higher levels of allelic diversity than that found in wild populations (Table 2). The *NA* was significantly higher in captive-born gorillas than in Virungas. For measures of *AR*, the captive-born gorillas were significantly higher than Virungas, Bwindi, Cross River and WWLG populations. Cross River and Virungas populations had significantly lower *AE* than captive-born. Captive-born gorillas had lower levels of *AR* than the wild-born founders. Captive-born *AR* was significantly lower than the wild born founders but *NA* was not significantly different. Effective alleles in captive born gorillas were also not significantly lower than founders.

**Table 2:** Mean measures of allelic diversity and heterozygosity

Population	*NA*	*AR*	*AE*	*H*_I_	*H*_O_	*H*_E_
Captive	7.25	7.03	4.63	0.86	0.87	0.76
Founder^1^	8.50	8.48	5.18	0.72	0.73*	0.80
Wild western Lowland gorillas^2^,^3^	6.62	5.82*	3.88	0.78	0.75*	0.68
Cross River^4^	6.00	4.91**	3.30*	0.68	0.68**	0.68
Bwindi^5^	6.13	5.06**	3.29*	0.71	0.70**	0.68
Virungas^6^	5.13*	4.22**	2.69*	0.71	0.68**	0.62*

*NA*, number of alleles; *AR*, allelic richness; *AE*, number of effective alleles; *H*_I_, individual heterozygosity; *H*_O_, observed heterozygosity; *H*_E_, Nei's expected heterozygosity. Significant values shown in bold.

1 Nsubuga et al. (2010).

2 Bradley et al. (2004), Bradley et al. (#b^900^).

3 Arandjelovic et al. (2010).

4 Bergl et al. (2008).

5 Nsubuga et al. (2008).

6 Bradley et al. (2005).

* *P* < 0.05.

** *P* < 0.005.

Captive-born gorillas had higher levels of two heterozygosity measures than the wild-born founders. The *H*_o_ was higher in the captive-born gorillas than in the founders. Captive-born gorillas also had significantly higher *H*_o_ than wild populations. Similarly, *H*_e_ was significantly higher in the captive-born gorillas than in Virungas. Captive-born gorillas did not significantly differ from the founders in *H*_e_.

### Population structure and assignment

**Figure 3:**
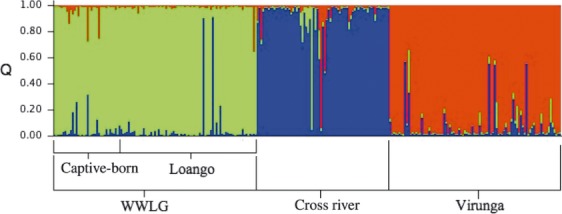
Structure results including captive-born and wild populations of western lowland gorillas, Cross-River gorillas, and mountain gorillas.

Comparisons of the second order rate of change, *ΔK* (Fig. 3) of the ln *P*(*D*) from STRUCTURE found a major breakpoint in the data with the highest likelihood of clusters at *K* = 3 (Pritchard et al. 2000; Evanno et al. 2005). The captive-born population clustered with the Loango population with the majority of individuals having a proportional group membership value (Q) > 80% (Fig. 3) at *K* = 3. Because captive gorillas and Loango gorillas are the same subspecies, we expected them to cluster together, which resulted in a *K* = 3, when combined with Cross River and Virunga populations.

**Figure 4:**
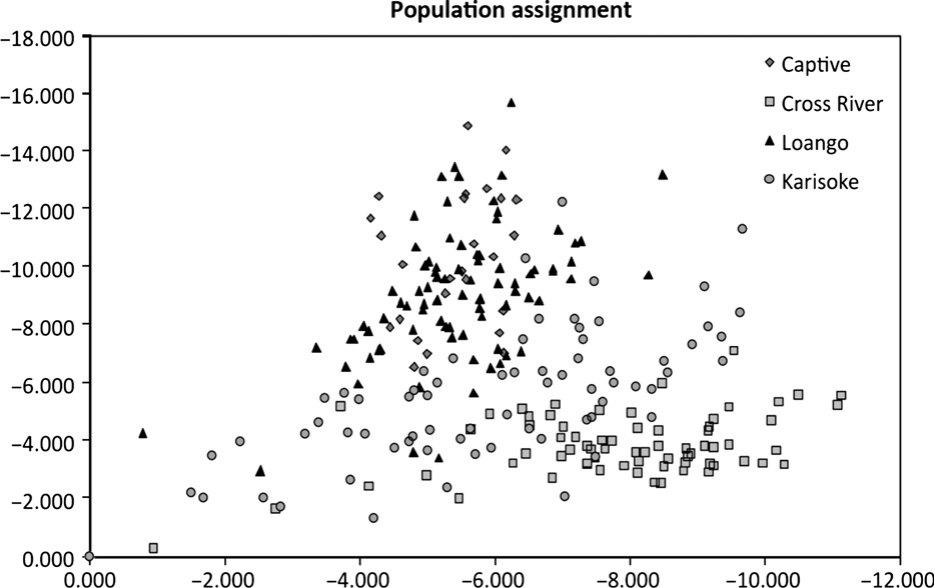
Frequency based population assignment of four populations including captive-born, Cross River, Loango and Virunga (Karisoke) gorillas.

**Figure 5:**
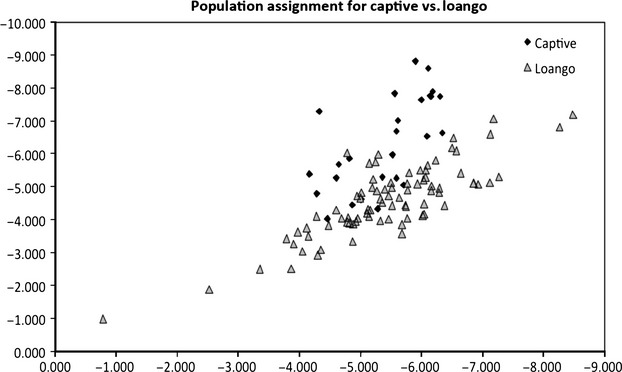
Pairwise frequency based population assignment plot including captive-born and Loango gorillas.

**Figure 6:**
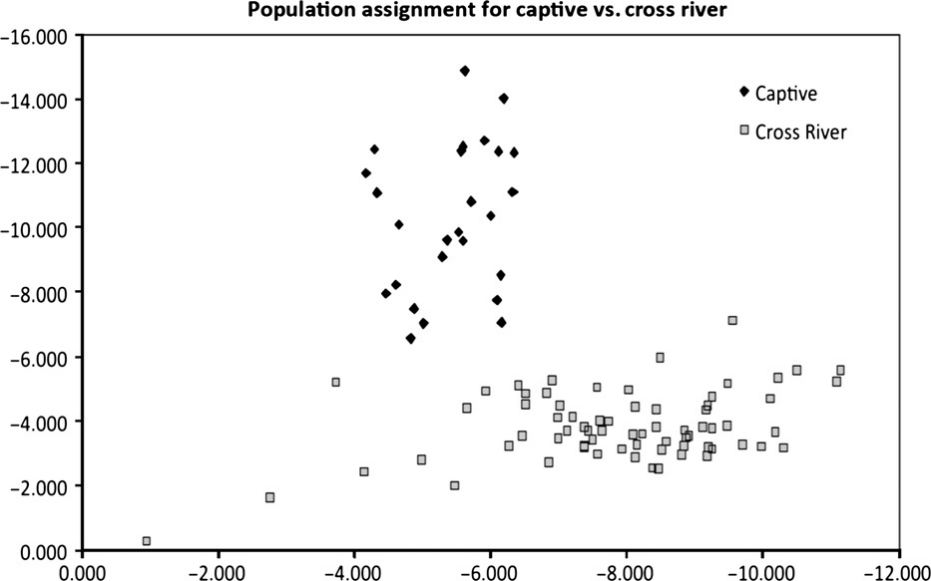
Pairwise frequency based population assignment plot including captive-born and Cross River gorillas.

**Figure 7:**
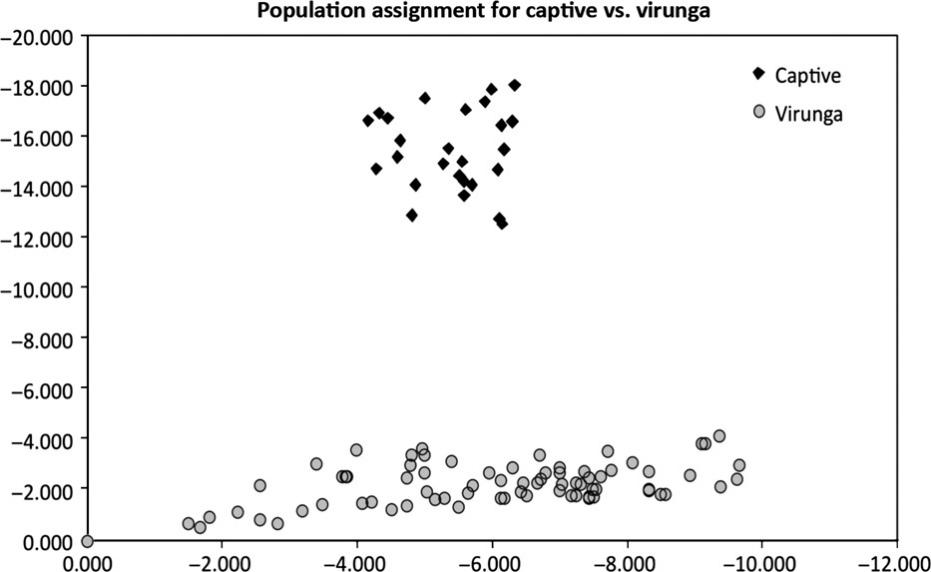
Pairwise frequency based population assignment plot including captive-born and Virunga gorillas.

Frequency based assignment tests agreed with the Structure results and clustered captive-born and Loango populations together to the exclusion of Cross River and Virungas (Fig. 4) when all four populations were included. Pairwise population assignments showed captive-born and Loango gorillas formed less discrete clusters than captive-born and other populations (Figs. 5–7). As the source for the captive populations are thought to come from Cameroon, the Congo and Gabon, we would expect that captive-born and Loango gorillas would cluster more closely than other populations to captive-born, which they do in the pairwise assignment, suggesting the methodology and markers used are accurate in assigning individuals to these populations.

### Genetic bottleneck signatures

The results of the BOTTLENECK analysis differed based on model choice but did not suggest the occurance of a bottleneck. The BOTTLENECK results using the TPM model were not significant according to any of the three tests, under any of the step-wise runs. Similarly, under a strict SMM, all three tests were not significant. In contrast, under a strict IAM, the Wilcoxon sign-rank test was significant for a one-tailed test of *H*_EQ_ excess (*P* = 0.002). The standardized differences test was also significant (*P* = 0.005). These results were not surprising as only a severe bottleneck would be detectable after only two generations. Further, if there were evidence of a bottleneck it would more likely reflect the demographic history of the source populations.

## Discussion

### Comparative genetic diversity of the captive-born population

Concerning the genetic diversity of the North American captive population of western lowland gorillas, they show high levels of both allelic diversity and heterozygosity. The captive-born population had a higher mean *NA*, *AR* and effective alleles than the wild populations. The captive-born population was significantly higher than all wild populations in mean *AR*, which is the most informative measure between these datasets. This meets the expectation that the captive-born gorillas would have high levels of allelic diversity when considering that the founder gorillas also have higher measures of allelic diversity than all wild populations. For all three measures, *NA*, *AR* and *AE*, the founder population had higher values than the captive-born, suggesting that while still high, the captive gorilla population has lost some allelic diversity present in the founder population. *H*_o_ was significantly higher in captive-born than founders, despite there being no significant difference in *H*_e_. This observation of decreased allelic diversity but increased *H*_o_ from the founder to captive-born population is interesting. We interpret this observation to be an artifact of changes to breeding protocols. Until recently breeding pairs were determined based on flawed pedigrees and closely related individuals were known to have bred (Nsubuga et al. 2010). This could have resulted in the loss of rare alleles, which may account for the decrease in allelic diversity from the founder to captive-born population. Recently, breeding based on non-random negative assortative mating according to least mean kinship between pairs would result in an increase in *H*_o_ from the founders, while *H*_e_ (which is based on *NA*) and *NA* have not had enough time to rebound through mutation. For measures of both allelic diversity and heterozygosity, the captive population is higher than all wild populations, despite the having lost allelic diversity since being in captivity.

### Genetic structure and population assignment

Based on the results of the STRUCTURE analysis, captive-born gorillas form two clusters, which is consistent with the clustering of the wild-born founders (Nsubuga et al. 2010). Structure results also found that when grouped with the Cross River, Loango and Virunga populations, the captive-born gorillas clustered with the Loango gorillas for a highest likelihood of three clusters. Assignment tests agreed with structure results that the captive-born and Loango gorillas were clustered together to the exclusion of Cross River and Virunga gorillas, and pairwise assignment test showed Loango and captive-born gorilla clustering more closely than captive-born and Cross-River or Virunga.

### Genetic bottleneck signatures

All tests under both a TPM and strict SMM were not significant. Results from both the Wilcoxon sign-rank test and standardized differences test under a strict IAM were significant, yet the IAM is the least conservative mutation model. The SMM, and to a greater degree, the TPM are considered more informative models in addressing questions of demographic history. These results suggest that there is no genetic evidence for a population bottleneck in this captive collection. While the population has lost allelic diversity, enough diversity has been maintained in the population that they are still significantly higher than comparable wild populations for several diversity measures. These results also suggest that despite flaws in pedigree information on founder gorillas, genetic management of this population has been successful in maintaining high levels of genetic diversity.

### Implications for conservation

There is currently no scientific justification for the use of the North American captive gorilla population as a source for reintroduction of individuals to the wild (Beck et al. 2007). As such, there is currently only one captive breeding program outside of a range country that re-introduces captive-born gorillas to the wild. The goal of the gorilla SSP is to manage the captive population of North American gorillas as single unit, and our results confirm those of Nsubuga et al. (2010) that the captive population should be managed as a single population. Measures of genetic diversity in the captive gorilla population are high compared to other captive mammal populations. The data presented here may be useful in the continued monitoring of genetic diversity in the captive population for the long-term maintenance of zoo collections. The data further suggests that the set of eight microsatellite loci used here represent an ideal panel for genetic management use; they are highly polymorphic, relatively neutral when accounting for closely related individuals in the sample and are all tetranucleotide repeat motif. In addition, as presented here, there exists a number of comparative wild datasets using this panel of loci, which can be used to estimate changes in genetic diversity over time in the captive population relative to wild populations.

## Data Accessibility

- File with genotype dataset.
- Readme.txt file for above referenced data file.

## Supporting Information

**Dataset S1.** North American captive-born gorilla genotype dataset.
